# Implementation of artificial intelligence in upper gastrointestinal endoscopy

**DOI:** 10.1002/deo2.72

**Published:** 2022-03-15

**Authors:** Sayaka Nagao, Yasuhiro Tani, Junichi Shibata, Yosuke Tsuji, Tomohiro Tada, Ryu Ishihara, Mitsuhiro Fujishiro

**Affiliations:** ^1^ Department of Gastroenterology Graduate School of Medicine the University of Tokyo Tokyo Japan; ^2^ Department of Endoscopy and Endoscopic Surgery Graduate School of Medicine the University of Tokyo Tokyo Japan; ^3^ Department of Gastrointestinal Oncology Osaka International Cancer Institute Osaka Japan; ^4^ Tada Tomohiro Institute of Gastroenterology and Proctology Saitama Japan; ^5^ AI Medical Service Inc. Tokyo Japan; ^6^ Department of Surgical Oncology Graduate School of Medicine the University of Tokyo Tokyo Japan

**Keywords:** adenocarcinoma of the esophagus, artificial intelligence, esophageal squamous cell carcinoma, pharyngeal neoplasms, stomach neoplasms

## Abstract

The application of artificial intelligence (AI) using deep learning has significantly expanded in the field of esophagogastric endoscopy. Recent studies have shown promising results in detecting and differentiating early gastric cancer using AI tools built using white light, magnified, or image‐enhanced endoscopic images. Some studies have reported the use of AI tools to predict the depth of early gastric cancer based on endoscopic images. Similarly, studies based on using AI for detecting early esophageal cancer have also been reported, with an accuracy comparable to that of endoscopy specialists. Moreover, an AI system, developed to diagnose pharyngeal cancer, has shown promising performance with high sensitivity. These reports suggest that, if introduced for regular use in clinical settings, AI systems can significantly reduce the burden on physicians. This review summarizes the current status of AI applications in the upper gastrointestinal tract and presents directions for clinical practice implementation and future research.

## INTRODUCTION

In recent years, the application of artificial intelligence (AI) technology using deep learning, especially convolutional neural network technology, is expanding in various medical fields. A similar trend is seen in the field of gastrointestinal (GI) endoscopy. AI systems for detecting colorectal polyps are commercially available in Japan, the United States, and some European countries. In addition, AI for detecting early esophageal cancer in Barrett's esophagus (BE) has been commercialized and is scheduled to be released in European countries. The world is collectively moving the stage of developing AI systems to the stage of implementing them.

 In this literature review, we discuss the latest findings from papers on convolutional neural network‐based imaging AI for detecting and diagnosing gastric, esophageal, and pharyngeal cancers. In addition, we discuss the role of AI in diagnosing Helicobacter pylori (*H. pylori*) gastritis and the anatomical classification of the stomach based on endoscopic images. Based on these research papers, we discuss the prospects of endoscopic diagnosis using AI in the field of upper GI tract endoscopy. 

## AI FOR DETECTION OF GASTRIC CANCER

Gastric cancer is one of the major cancer types diagnosed globally and is the third leading cause of cancer‐related deaths worldwide.^1^ Even in Japan, where mass screening for gastric cancer has long been established, the 5‐year overall survival rate of node‐negative early gastric cancer with stage IA is reported to be 91.5%.^2^ Therefore, early detection and treatment of gastric cancer are mandatory. Endoscopy plays an important role in diagnosing and treating early gastric cancer; endoscopic diagnosis is imperative, and endoscopic submucosal dissection (ESD) is widely used to treat early gastric cancer.^3^, ^4^ In recent years, studies have reported the effectiveness of endoscopy using AI support systems (Table 1).

**TABLE 1:** Summary of artificial intelligence for diagnosing in stomach field

Name (year)^Ref^	Study design	Imaging modality	Training dataset (images)	Test dataset (images)	AUC	Accuracy (%)	Sensitivity (%)	Specificity (%)
**Detection**								
Hirasawa T (2018)^6^	Retrospective	WLI	Abnormal 13,584	2296	n/a	n/a	92.2	n/a
Sakai Y (2018)^68^	Retrospective	WLI	58 patients	58 patients	0.958	87.6	80	94.8
Ishioka M (2019)^7^	Retrospective	WLI	Abnormal 13,584	68 videos	n/a	n/a	94.1	n/a
Wu L (2019)^69^	Retrospective	WLI, NBI, BLI	9151 abnormal 3170	200	n/a	92.5	94	91
Yoon HJ (2019)^21^	Retrospective	WLI	11,539 (abnormal 1705)	0.981	n/a	91	97.6	
Luo H (2019)^9^	Multicenter, case‐control (including esophageal cancer)	WLI	141,570 (abnormal 35,531)	66750 (abnormal 4317)	0.974	92.7	94.6	92.6
Tang D (2020)^70^	Retrospective	WLI	35,823 (abnormal 26,172)	9417 (abnormal 4153)	0.94	87.8	95.5	81.7
Ikenoyama Y (2021)^8^	Retrospective	WLI	Abnormal 13,584	2940 (abnormal 209)	0.757	n/a	58.4	87.3
Wu L (2021)^10^	Randomized controlled trial	WLI, NBI, BLI	7321 (abnormal 2530)	302,692	n/a	84.7	100	84.3
***H.pylori* infection**								
Huang CR (2004)^71^	Prospective	WLI	30 patients	74 patients	n/a	n/a	85.4	90.9
Shichijo S (2017)^11^	Retrospective	WLI	32,208	11,481	0.93	87.7	88.9	87.4
Itoh T (2018)^72^	Prospective	WLI	149	30	0.956	n/a	86.7	86.7
Nakashima H (2018)^73^	Prospective	WLI, BLI‐bright, LCI	162 patients	60 patients	0.66 (WLI) 0.96 (BLI‐bright) 0.95 (LCI)	n/a	66.7 (WLI) 96.7 (BLI‐bright) 96.7 (LCI)	60 (WLI) 86.7 (BLI‐bright) 83.3 (LCI)
Shichijo S (2019)^12^	Retrospective	WLI	98,564	23,699	n/a	80(*H. pylori*‐negative) 48(‐positive) 84(‐eradicated)	n/a	n/a
Zheng W (2019)^74^	Retrospective	WLI	11,729	3755	0.97	93.8	91.6	98.6
Guimarães P (2020)^75^	Retrospective	WLI	200	70	0.981	92.9	100	87.5
Yasuda T (2020)^76^	Retrospective	LCI	32 patients	105 patients	n/a	87.6	90.5	85.7
Zhang Y (2020)^77^	Retrospective	WLI	5470	0.99	94.24	94.58	94.01	
Nakashima H (2020)^13^	Prospective	WLI, LCI	12,887	120 videos	0.90 (LCI, uninfected) 0.82 (LCI, currently infected) 0.77 (LCI, post‐eradication)	75.0 (WLI, uninfected) 84.2 (LCI, uninfected) 77.5 (WLI, currently infected) 82.5 (LCI, currently infected) 74.2 (WLI, post‐eradication) 79.2 (LCI, post‐eradication)	95.0 (WLI, uninfected) 92.5 (LCI, uninfected) 60.0 (WLI, currently infected) 62.5 (LCI, currently infected) 35.0 (WLI, post‐eradication) 65.0 (LCI, post‐eradication)	65.0 (WLI, uninfected) 80.0 (LCI, uninfected) 86.2 (WLI, currently infected) 92.5 (LCI, currently infected) 93.8 (WLI, post‐eradication) 86.2 (LCI, post‐eradication)
Xu M (2021)^78^	Prospective	ME‐NBI, ME‐BLI	354 patients	77 patients	0.878	87.8	96.7	73
**Invasion depth**								
Kubota K (2012)^79^	Retrospective	WLI	902	n/a	64.7	n/a	n/a	
Zhu Y (2019)^20^	Retrospective	WLI	790	203	0.94	89.16	76.47	95.56
Yoon HJ (2019)^21^	Retrospective	WLI	1705	0.851	n/a	79.2	77.8	
Nagao S (2020)^22^	Retrospective	WLI, NBI, indigo‐carmine dye contrast imaging	13,628	2929	0.9590 (WLI) 0.9048 (NBI) 0.9491 (indigo‐carmine dye contrast imaging)	94.49 (WLI) 94.30 (NBI) 95.50 (indigo‐carmine dye contrast imaging)	84.42 (WLI) 75.00 (NBI) 87.50 (indigo‐carmine dye contrast imaging)	99.37 (WLI) 100 (NBI) 100 (indigo‐carmine dye contrast imaging)
Cho BJ (2020)^80^	Retrospective	WLI	2899	206	0.887	77.3	80.4	80.7
Tang D (2021)^81^	Retrospective	WLI	3407	228	0.942	88.16	90.48	85.29

Abbreviations: AUC, area under the curve; BLI, blue laser imaging; *H. pylori, Helicobacter pylori*; LCI, linked color imaging; ME, magnifying endoscopy; NBI, narrow‐band imaging; WLI, white light imaging.

Although it is known that endoscopy helps detect gastric cancer early, a meta‐analysis revealed that the missed rate of upper GI cancer is 6.4% and 11.3% within 1 and 3 years, respectively, before diagnosis, indicating a certain probability of missed cases.^5^ To reduce the number of missed cases to the maximum extent and to detect early gastric cancer with stable performance, researchers have developed an AI support system to detect gastric cancer in recent years. In 2018, Hirasawa et al. developed a gastric cancer detection AI using 13,584 gastric cancer images as a training set. The gastric cancer diagnostic ability for a test set of 2296 images showed a very high sensitivity of 92.2%.^6^ In addition, they demonstrated that the developed AI system achieved a sensitivity as high as 94.1% using video images of 68 lesions.^7^ According to a report comparing gastric cancer diagnosis rates of AI against endoscopists, AI showed a sensitivity of 58.4%, exceeding that of endoscopists (31.9%).^8^ These results imply that using an AI support system might improve the detection rate of gastric cancer. A multicenter, case‐control study conducted by Luo et al., in 2019, to evaluate gastric and esophageal cancers showed an accuracy of 92.7% for cancer detection in the prospective validation set.^9^

Wu et al. developed an AI system to reduce the number of blind spots and detect gastric cancer (ENDOANGEL) and conducted a randomized controlled study to verify its diagnostic effectiveness. In their study, AI achieved an accuracy of 84.7%, sensitivity of 100%, and specificity of 84.3% for detecting gastric cancer, demonstrating that the diagnostic ability of the AI‐assisted endoscopy group was better than that of the control group.^10^

Several reports have suggested the effectiveness of AI‐assisted endoscopy for the early detection of gastric cancer. These reports might accelerate the adoption of AI‐based tools in real‐world clinical practice in the future.

## AI FOR DIAGNOSIS OF *H. PYLORI* INFECTION

*H. pylori* infection is one of the most critical risk factors for gastric cancer. Data mining the presence or absence of *H. pylori* infection by endoscopy can help identify the high‐ or low‐risk population for gastric cancer and contribute to the early diagnosis of gastric cancer. Shichijo et al., in 2017, reported the use of AI to detect the presence of *H. pylori* infection from gastric mucosal findings by endoscopy.^11^ The AI was trained using 32,208 images for the training set. Its discriminative ability to detect *H. pylori* infection was evaluated on a test set of 11,481 images. The accuracy of detecting *H. pylori* infection was found to be 87.7%, with a sensitivity of 88.9% and specificity of 87.4%. This indicated excellent diagnostic performance and superiority of detection to that of beginner endoscopists. In 2019, Shichijo et al. developed an AI system that could discriminate between *H. pylori*‐positive, *H. pylori*‐negative, and *H. pylori*‐eradicated using a training set of 98,564 images.^12^ The ability to discriminate among *H. pylori*‐positive, *H. pylori*‐negative, and *H. pylori*‐eradicated was evaluated on a test set of 23,699 images, with a diagnostic accuracy of 80% (*H. pylori*‐negative), 48% (*H. pylori*‐positive), and 84% (*H. pylori*‐eradicated), respectively. Nakashima et al. also evaluated the accuracy of *H. pylori* diagnosis using white light imaging (WLI) and linked color imaging (LCI), a type of equipment‐based image‐enhanced endoscopy (IEE).^13^ The accuracy of detection was found to be 75.0% (WLI, uninfected), 84.2% (LCI, uninfected), 77.5% (WLI, currently infected), 82.5% (LCI, currently infected), 74.2% (WLI, post‐eradication), and 79.2% (LCI, post‐eradication), respectively, indicating higher accuracy in LCI than in WLI. These studies suggest the usefulness of AI support systems in diagnosing *H. pylori* infection. Combining AI screening with IEE will be an interesting topic for exploration in the future.

## AI FOR DIAGNOSIS OF THE INVASION DEPTH OF GASTRIC CANCER

Since the 2000s, ESD has been developed as an improved version of endoscopic mucosal resection.^14^ The development of ESD has made it possible to perform en bloc resection of many lesions regardless of the presence of ulcer scars or the size of the lesion and achieve a good long‐term prognosis comparable to surgical treatment.^15^, ^16^ It allowed clinicians to investigate the risk of lymph node metastasis in surgically resected gastric cancer, thereby expanding the range of lesions amenable to ESD. This further established ESD as a minimally invasive and curative treatment for early gastric cancer.^17^, ^18^ ESD is an excellent treatment method that preserves organs and ensures the patient's quality of life in terms of early recovery of pain and function and subsequent appetite and nutrition.^19^ Among the several factors, including histological type, tumor size, presence or absence of lymphovascular infiltration, and presence or absence of ulcerative findings, invasion depth is an essential factor in determining the curability of ESD.

In most cases of intramucosal cancer (M cancer) and < 500 μm from the muscularis mucosae cancer (SM1 cancer), follow‐up after ESD is acceptable. However, additional surgical resection is needed for submucosal invasive cancer deeper than 500 μm (SM2 cancer). Therefore, discriminating between M‐SM1 cancer and cancer deeper than SM2 is an essential criterion in determining the treatment strategy for gastric cancer. In recent years, AI tools have been used to diagnose the invasion depth of gastric cancer.

**FIGURE 1:**
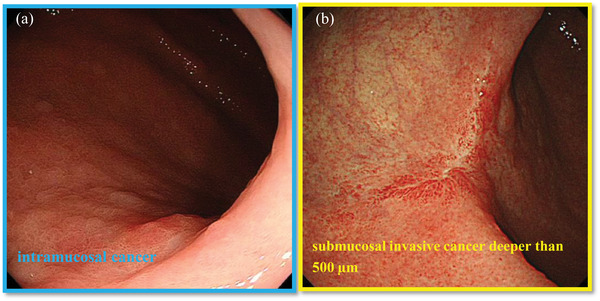
Gastric cancer depth prediction using artificial intelligence (AI) support system. (a) The AI support system correctly predicted intramucosal cancer. (b) The AI support system correctly predicted submucosal invasive cancer deeper than 500 μm

Zhu et al. assessed the efficacy of AI tools for assessing invasion depth of gastric cancer (M‐SM1 vs. SM2 or deeper). They observed a sensitivity of 76.5%, specificity of 95.6%, and an accuracy of 89.2%, with higher accuracy and specificity than endoscopists.^20^ Yoon et al. also investigated the same topic and reported a sensitivity of 79.2% and specificity of 77.8% for invasion depth.^21^ Nagao et al. reported that their AI system accurately predicted the invasion depth of gastric cancer (M‐SM1 vs. SM2 or deeper), with a sensitivity per lesion of 84.4%, specificity of 99.4%, and accuracy of 94.5% (Figure 1).^22^ Nagao et al. also evaluated the diagnostic ability of AI systems dedicated to narrow‐band imaging (NBI) and indigo‐carmine dye contrast imaging. They found that in NBI, the sensitivity, specificity, and accuracy per lesion were 75.0%, 100.0%, and 94.3%, respectively. For indigo‐carmine dye contrast imaging, the sensitivity, specificity, and accuracy per lesion were 87.5%, 100.0%, and 95.5%, respectively. There were no significant differences among the three AI systems in terms of diagnostic ability. These reports suggest that the AI support system may be helpful to detect invasion depth. It must be verified whether the prediction is more accurate when the AI system is combined with an endoscopist's guidance in real‐world clinical practice. Improving the accuracy of AI‐supported diagnosis of invasion depth can help select the most appropriate treatment improving the standard of care for all the patients.

## AI FOR DIAGNOSIS OF ESOPHAGEAL SQUAMOUS CELL CARCINOMA

Esophageal cancer is the seventh most common cancer and the sixth most common cause of cancer‐related mortality worldwide.^1^ Squamous cell carcinoma is the predominant type of esophageal cancer in Asia, Africa, and South America.^23^ The prognosis for advanced esophageal squamous cell carcinoma (ESCC) is poor. However, if detected at an early stage and resected endoscopically, a favorable prognosis can be expected.^24^ IEE, such as NBI, helps detect early ESCC.^25^ However, the same can be challenging for less experienced endoscopists.^26^ Experienced endoscopists may miss early ESCC due to several reasons, including physical condition and carelessness. As a result, patients with missed early ESCC can lose the opportunity for endoscopic treatment. In such cases, an AI system can potentially reduce the chances of early ESCC being overlooked due to human factors.

**FIGURE 2:**
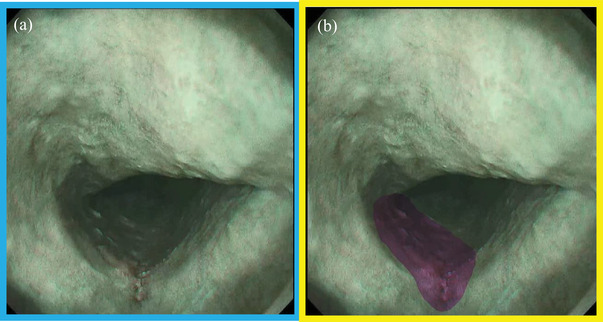
Detection of esophageal squamous cell carcinoma (ESCC) by artificial intelligence (AI) system. (a) The lesion was brownish and slightly depressed in narrow‐band imaging. (b) The lesion was indicated in pink by the AI system

The usefulness of AI in detecting and characterizing ESCC has already been reported in many studies (Table 2).^27^, ^28^, ^29^, ^30^, ^31^, ^32^, ^33^, ^34^, ^35^ Several studies have used video images as validation sets,^28^, ^29^, ^31^, ^33^, ^35^ which is more realistic and challenging than still images. Waki et al.^34^ evaluated the detection of an AI system using 100 video images (Figure 2). In this study, the AI system had high sensitivity (85.7%, 54 of 63 early ESCCs) for detecting ESCC and increased endoscopists’ sensitivity without reducing specificity. Shiroma et al.^33^ evaluated the efficiency of an AI system using slow‐ and high‐speed video images. The sensitivity of the AI system was 100% (32 of 32 early ESCCs) in the slow‐speed videos and 85% (17 of 20 cases) in the high‐speed videos. Moreover, the sensitivity of endoscopists improved with the real‐time assistance of the AI diagnostic system. These studies were unique in such a manner that the validation video images were captured by passing the endoscope through the esophagus at a constant speed without focusing on the lesions or any particular parts to simulate the situation of overlooking ESCC.

**TABLE 2:** Summary of artificial intelligence in the detection of early esophageal squamous cell carcinoma (ESCC) with non‐magnified endoscopy

**Name (year)^Ref^**	**Study design**	**Histology of cases**	**AI algorithm**	**Endoscopic images**	**Data category**	**Number of cases in test dataset**	**Number of controls in test dataset**	**TP**	**FP**	**FN**	**TN**
Cai (2019)^27^	Retrospective	ESCC/HGIN/LGIN	CNN	WLI	Still images	91 images	96 normal images	89	14	2	82
Fukuda (2020)^28^	Retrospective	ESCC	CNN	NBI/BLI	Video images	45 ESCCs	99 normal and noncancerous lesions	41	48	4	51
Ohmori (2020)^30^	Retrospective	ESCC	CNN	WLI	Still images	52 ESCCs	83 normal and noncancerous lesions	47	20	5	63
				NBI/BLI	Still images	52 ESCCs	83 normal and non‐cancerous lesions	52	31	0	52
Yang (2020)^31^	Retrospective	ESCC	CNN	WLI/OE/ Iodine stain	Still images	76 ESCCs	780 normal/benign lesions	74	11	2	769
				WLI/OE	Video images	20 ESCCs	28 video images of normal esophagus	19	2	1	26
Li (2021)^32^	Retrospective	ESCC/HGIN/LGIN	CNN	WLI/NBI	Still images	266 images	366 normal images	252	37	14	329
Shiroma (2021)^33^	Retrospective	ESCC	CNN	WLI	Video images	20 ESCC patients	20 patients without ESCC	15	14	5	6
				NBI	Video images	20 ESCC patients	20 patients without ESCC	11	4	9	16
Waki (2021)^34^	Retrospective	ESCC	CNN	NBI/BLI	Video images	63 ESCCs (50 video images)	50 video images of normal and noncancerous lesions	54	30	9	20
Wang (2021)^35^	Retrospective	ESCC/HGD/LGD	CNN	WLI/NBI	Still images	210 images	54 images of normal esophagus	202	16	8	38

Abbreviations: AI, artificial intelligence; BLI, blue‐laser imaging; CNN, convolutional neural network; ESCC, esophageal squamous cell carcinoma; FN, false negative; FP, false positive; HGD, high‐grade dysplasia; HGIN, high‐grade intraepithelial neoplasia; LGD, low‐grade dysplasia; LGIN, low‐grade intraepithelial neoplasia; NBI, narrow‐band imaging; OE, optical enhancement; TN, true negative; TP, true positive; WLI, white‐light imaging.

An accurate diagnosis of the invasion depth is essential when determining the treatment strategy for ESCC because clinically diagnosed epithelium (EP)/lamina propria mucosa (LPM) and muscularis mucosa (MM)/submucosal cancers invade up to 200 μm (SM1) are indication for endoscopic resection^82^. In contrast, esophagectomy or chemoradiotherapy is mainly indicated for SM2‐3 ESCC.^36^, ^37^ Magnified endoscopy (ME) and endoscopic ultrasonography are preferable to non‐ME for diagnosing invasion depth in ESCC.^38^ However, extensive knowledge and experience are essential to master these modalities. Furthermore, evaluating the invasion depth using these techniques is susceptible to interobserver differences. Objective evaluation using a high‐performance AI system may help less experienced endoscopists, as well as experienced endoscopists, reach an appropriate diagnosis.

There are several reports on the diagnosis of the invasion depth of superficial ESCC using AI. Tokai et al.^39^ developed an AI system to distinguish EP‐SM1 ESCC from deeper than SM2 ESCC with non‐ME still images. The accuracy was found to be 80.9%, with an AUC greater than 13 board‐certified endoscopists. Nakagawa et al.^40^ developed an AI system to distinguish EP‐SM1 ESCC from SM2‐3 ESCC with non‐ME and ME still images. The accuracy was found to be 91.0%, with a performance similar to 16 experienced endoscopists. Shimamoto et al.^41^ developed an AI system to distinguish EP‐SM1 from SM2‐3 in superficial ESCC using 102 video images consisting of two types: non‐ME with WLI and ME with NBI/blue‐laser imaging. The accuracy of the AI system in non‐ME videos and ME videos was found to be 87.3% and 89.2%, respectively, higher than 14 board‐certified endoscopists.

## AI FOR DIAGNOSIS OF ESOPHAGEAL ADENOCARCINOMA

Esophageal adenocarcinoma (EAC) is the predominant esophageal cancer in North America and Europe.^23^ BE is a known risk factor for EAC, and endoscopic surveillance of BE is recommended.^42^ Advanced EAC requires invasive treatment and has a poor prognosis. In contrast, T1 EAC can be cured with less invasive endoscopic treatment.^43^, ^44^ Early detection is vital to reduce mortality related to EAC. However, early detection remains a challenging task for non‐experts.^45^ An AI tool could possibly support the endoscopic diagnosis of EAC.

Several studies on the AI system for diagnosing early EAC have been reported in the West^46^, ^47^, ^48^, ^55^, and a few of them were about real‐time diagnosis (Table 3).^46^, ^47^ de Groof et al.^48^ developed an AI system to detect Barrett's neoplasia, which achieved accuracy higher than any of the 53 endoscopists. Furthermore, this AI system detected Barrett's neoplasia with high accuracy during live endoscopic procedures in a prospective pilot study.^46^ Ebigbo et al.^47^ developed an AI system to capture random images from a real‐time camera and differentiate between normal BE and early EAC; the sensitivity, specificity, and accuracy of this system were 83.7%, 100.0%, and 89.9%, respectively. These studies highlighted the usefulness of AI systems for early EAC. However, most of the studies were performed in Western countries. The characteristics of EAC were different in the West and Asia^49^; therefore, it is questionable whether the AI system developed using the training set based on Western cases is acceptable for clinical practice in Asia. As the number of EACs in Asia is suggested to increase over coming years,^50^ developing an AI system trained with EAC cases in Asia is imperative. Iwagami et al.^51^ developed an AI system based on Japanese cases to detect esophageal and esophagogastric junctional adenocarcinoma. They observed a sensitivity, specificity, and accuracy of 94%, 42%, and 66%, respectively.

**TABLE 3:** Summary of artificial intelligence in the detection of early esophageal adenocarcinoma (EAC) with non‐magnified endoscopy

**Reference (year)**	**Study design**	**Histology of cases**	**AI algorithm**	**Endoscopic images**	**Data category**	**Number of cases in test dataset**	**Number of controls in test dataset**	**TP**	**FP**	**FN**	**TN**
de Groof (2020)^48^	Retrospective	EAC/HGD	CNN	WLI	Still image	209 images	248 images of non‐dysplastic BE	186	31	23	217
de Groof (2020)^46^	Prospective	EAC/HGD	CNN	WLI	Still image	33 images	111 images of non‐dysplastic BE	25	15	8	96
Hashimoto (2020)^55^	Retrospective	EAC/HGD	CNN	WLI(+near focus)	Still image	146 images	79 images of non‐dysplastic BE	144	12	2	95
				NBI(+near focus)	Still image	79 images	126 images of non‐dysplastic BE	73	1	6	125
Iwagami (2021)^51^	Retrospective	EAC(EGJ)	CNN	WLI/NBI/BLI	Still image	36 EACs	43 non‐cancerous	34	25	2	18

Abbreviations: AI, artificial intelligence; BE, Barrett's esophagus; BLI, blue‐laser imaging; CNN, convolutional neural network; EAC, esophageal adenocarcinoma; EGJ, esophagogastric junction; FN, false negative; FP, false positive; HGD, high‐grade dysplasia; NBI, narrow‐band imaging; TN, true negative; TP, true positive; WLI, white‐light imaging.

## AI FOR DETECTION OF PHARYNGEAL CANCER

Pharyngeal cancer has a poor prognosis because it is often detected at an advanced stage. Patients with advanced pharyngeal cancer require surgery and chemoradiotherapy, which decreases their quality of life. On the other hand, patients with superficial pharyngeal cancer (SPC) can be cured by endoscopic resection, which is less invasive than surgery and chemoradiotherapy. IEE, such as NBI, can help detect SPC.^25^ However, it is challenging to perform for less experienced endoscopists. An AI system can possibly improve the detection of SPCs in such cases.

Tamashiro et al.^52^ evaluated the AI system using 1912 still images from 35 patients with 40 pharyngeal cancers and 40 patients without pharyngeal cancer. The AI system detected all pharyngeal lesions, and the sensitivity and specificity per image were 79.7% and 57.1%, respectively. Kono et al.^53^ evaluated an AI system using 25 video images of pharyngeal cancer and 36 video images of non‐pharyngeal cancer. In this study, the sensitivity, specificity, and accuracy for detecting cancer were 92%, 47%, and 66%, respectively.

## FUTURE PROSPECTS

The development of AI in the gastric region has progressed significantly, and it is expected to be introduced into real‐world clinical practice in the near future. With the help of diagnostic support from AI tools, trainee endoscopists might be able to reach endoscopic diagnoses similar to expert endoscopists, regardless of their skill level. The use of AI in clinical practice remains an important issue. For example, it remains to be determined whether diagnosis using movies or still images is better for AI‐assisted endoscopy. While real‐time diagnosis is essential for detection, still images might be considered appropriate when detecting *H. pylori* infection and invasion depth in clinical practice. In addition, it is necessary to investigate how many functions should be included in a single AI system for clinical use in the future.

The usefulness of the AI system in diagnosing ESCC has been reported in many studies. However, there are several problems associated with its use in clinical practice. Most of these studies are single‐center retrospective studies, and the images used in validation sets are edited to some extent; therefore, selection bias cannot be ruled out. Well‐designed prospective studies in a multicenter setting are required. The specificity of the AI system for detecting ESCC in studies using video images as a validation set remains very low. This is a further problem in clinical practice because the proportion of ESCC patients in the validation set is higher than in the real world, and, therefore, the positive predictive value would considerably decrease in clinical practice. One of the strategies to solve this problem is to use a combination of two AI systems: a sensitivity‐oriented AI system with non‐ME that focuses on detection and an accuracy‐oriented AI system with ME that focuses on characterization. Although further improvement of the AI system and prospective studies in a multicenter setting is needed, we believe that coming years will witness the use of AI systems for ESCC diagnosis.

There are many reports on the usefulness of AI systems for diagnosing EAC, and the AI system will soon help endoscopists diagnose early EAC. However, there are several concerns with its use in clinical practice, such as ESCC. Most of these cases were retrospective studies, and the number of cases in the validation sets was small. Prospective studies with a larger number of cases in a multicenter setting are needed to obtain a better and more accurate algorithm. In these AI systems, still images were used as validation sets. Because the length of BE is short, the AI system based on still images may be helpful in clinical practice. However, an AI system based on video images may be more appropriate for detecting EAC, as it may reduce the chances of overlooking lesions as against an AI trained on pictures with poor quality.

Tamashiro et al.^52^ and Kono et al.^53^ showed high sensitivity in AI‐based diagnosis; however, the performance of AI in terms of specificity was not satisfactory. As Kono et al. mentioned, the complicated structure of the pharyngeal area and poor observation conditions due to the presence of saliva, mucus, or gag reflux might affect specificity, and further training with cancer images and normal structural images under various conditions is required to improve the specificity.^53^ An AI system with magnified endoscopic images for characterization may also improve the specificity.^54^ However, it is difficult to accumulate sufficient SPC cases in a single institution. It is necessary to train and evaluate an AI system with more SPC and normal structural images from multiple facilities for practical use in clinical practice.

### Implementation of AI systems in upper GI endoscopy

AI tools for endoscopic devices, especially for the lower GI tract, have already been certified by regulatory authorities in various countries. Several companies have commercialized AI devices for the real‐time detection of colorectal polyps in Europe. The device authorized for marketing by the US Food and Drug Administration, which uses AI to detect colon polyps and suspected colon tumors in real‐time has been commercialized. In addition, AI devices to detect colorectal polyps and those to differentiate colorectal polyps and to evaluate ulcerative colitis using super‐magnifying endoscopes have been approved by regulatory authorities in Japan.

However, there are few authorized AI products for the upper GI tract. AI tools for detecting neoplasia in BE have already obtained CE markings in Europe. However, there are no AI products certified by regulatory authorities to detect gastric cancer or neoplastic lesions of the stomach.

As this situation suggests, there are fewer randomized controlled trials and prospective studies on the upper GI tract^56^ than on the lower GI tract.^57^, ^58^, ^59^, ^60^, ^61^, ^62^, ^63^, ^64^ One possible reason for this is the difference in the difficulty of detecting lesions. It has been reported that the false‐negative rate of detection by gastroscopy is higher than that of detection by colonoscopy.^65^ Gastric cancer is difficult to recognize, unlike colorectal cancer, and may be overlooked even if the lesion is visible on endoscopic images. ESCC has been reported to be more difficult to detect with white light than with NBI and Lugol chromoendoscopy,^66^ which may also be a reason for fewer studies conducted. Moreover, differences in disease incidence by region may have influenced the decision to conduct a major clinical study. The incidence of gastric cancer is high in East Asia, corresponding to the high prevalence of H. pylori.^67^ There are two major histological types of esophageal cancer: ESCC and EAC. ESCC is more common in Asia, Africa, and South America, while EAC is more common in North America and Europe.^1^, ^23^

However, as described in this review, there have been various reports of AI systems for the upper GI tract, and it is expected that many products will emerge in the future that will be certified by the regulatory authorities.

## CONCLUSION

This review outlines recent research and the prospects of AI application for the endoscopic diagnosis of the upper GI tract. Unlike the detection of colorectal polyps, the early detection of upper GI cancers by AI can significantly impact prognosis, and its usefulness is highly anticipated. Employing AI‐based endoscopes is expected to enable early cancer detection and, consequently, improve patient prognosis. Due to the difference in diagnostic ability among endoscopists, either due to experience or subjective bias, using an AI tool as an accessory can help reduce the risk of overlooking malignant lesions and equalizing their diagnostic ability. An AI tool can recognize lesions in endoscopic images and determine their probability. However, it cannot perform endoscopy or reach a final diagnosis. Thus, the demand for digestive endoscopists will remain the same despite the introduction of AI tools. In the future, endoscopists will be required to understand the capabilities of AI and its handling and accordingly use endoscopes to navigate and observe the GI tract, including the pharynx.

## CONFLICT OF INTEREST

Tada T is a shareholder of AI Medical Service Inc. The authors have no other relevant affiliations or financial involvement with any organization or entity with a financial interest in or financial conflict with the subject matter or materials discussed in the manuscript apart from those disclosed.

## FUNDING INFORMATION

None.
